# Closing the Guideline Gap: AI and the Rise of Living Cardiovascular Recommendations

**DOI:** 10.1016/j.jacadv.2026.102639

**Published:** 2026-02-26

**Authors:** Rohan G. Reddy, David A. Danford, Andreas Schuster, Shelby Kutty

**Affiliations:** a NYU Grossman School of Medicine, New York, New York, USA; b BayCare Health System, Clearwater, Florida, USA; c Department of Cardiology and Pneumology, University Medical Center Göttingen, Georg-August University, Göttingen, Germany

**Keywords:** artificial intelligence, clinical practice guidelines, living clinical practice guidelines

Contemporary cardiology practice is shaped by rapid advances in diagnostics and therapeutics. The clinician is inundated with data from high-quality clinical trials that reveal the advantages of newly developed devices, pharmaceuticals, and novel care strategies. The expectation that this research will bring improved outcomes for patients requires its translation into evidence-based clinical practice guidelines (CPGs). These are generally produced every few years and are published only after completion of a multistep, time-intensive procedure. This timeline can be especially problematic in rapidly evolving areas such as management of heart failure, atrial fibrillation anticoagulation, and treatment of acute coronary syndrome. This leads to outdated CPGs, with physicians making difficult choices when the literature may be at odds with published guidance. For example, the median time lag is 2 years from the publication of pivotal clinical trials in acute coronary syndrome management to their incorporation into CPGs, and 16 years from publication of pivotal clinical trials to 90% uptake in clinical practice.^1^ Such intervals of misalignment between published data and CPG recommendations can lead to variability in care delivery, further expansion of disparities between high- and lower-resource centers, and uncertainty among physicians, thereby contributing to already suboptimal adoption of guideline-recommended therapies. Furthermore, when new recommendations are incorporated into CPGs, the level of evidence often evolves quickly. Analysis of successive European Heart Failure guidelines has shown shifts in the level of evidence for recommendations across versions, highlighting their dynamic nature even as they are incorporated into a CPG.^2^

Living CPGs (LCPGs) offer a rapid and efficient alternative to the relatively static CPGs currently in use. Unlike traditional CPGs, LCPGs allow individual recommendations to be updated in response to new evidence, enabling faster integration and dissemination of recommendations without the time-consuming process of revising the entire document. Thus, LCPGs effectively minimize the lag between the emergence of new data and its incorporation into guidelines.^3^ Cardiovascular medicine, with its robust evidence base, is uniquely positioned to benefit from this model. In fact, a 2012 survey by the American Heart Association found that more than two-thirds of cardiologists support dynamically updated CPGs when new data warrant changes to guideline recommendations.^4^ Despite general recognition of their advantages, implementation of LCPGs has lagged. The most commonly cited challenges to adoption of LCPGs are the lack of infrastructure for continuous evidence monitoring and updating, reliance on consensus panels, and the need to switch to fully digital guideline formats.^5^ However, advancements in artificial intelligence (AI) may overcome many of these barriers by enabling automated systematic reviews, providing machine-readable knowledge representation, and integrating guideline recommendations into electronic health record (EHR)-based clinical decision support (CDS) tools. We present here the potential benefits of LCPGs specifically in cardiology and describe how AI might serve to promote their successful implementation.

## Role of AI in living clinical practice guidelines

LCPGs are guidelines that are updated by revising individual recommendations in response to new relevant evidence as soon as it becomes available. The framework of LCPGs is the living systematic review, which requires continual monitoring of the literature to facilitate rapid updates in response to new evidence. Undertaken without technologic support, living systematic reviews would be very labor-intensive, but AI-based literature surveillance tools can significantly automate the process. For example, AI-based search and ingestion agents could reduce human workload by automating the review of journals, new trials, and databases from which the algorithms would extract relevant evidence.^6^ The product of this automated review would be living summary tables for review by human guideline panels, including an Evidence Profile and an Evidence to Decision table that provide key information on benefits, harms, evidence quality, equity, methodologic limitations, and generalizability of findings. Although establishing a living guideline panel and a living peer review process is necessary to maintain oversight and assure the integrity of recommendations, and requires human labor, the AI-supported review would greatly streamline the panel’s work.^6^

For the living systematic review and guideline development process to be effective, the process requires several built-in capabilities. First, AI must facilitate, through shared platforms and automated data transfers, integration of living systematic review teams with guideline development teams. Second, LCPGs, with their continual surveillance methodology, must avoid excessive triggering of updates in response to new low-certainty data.^6^ Therefore, establishing appropriate update thresholds is paramount for balancing data-responsiveness with caution. AI-driven statistical monitoring can be titrated to detect clinically meaningful effect-size shifts and flag only new evidence likely to alter practice. Third, for LCPGs to be effective, they must be easily and widely disseminated in an updatable format. Unlike the CPG, for which the PDF has been a suitable format, LCPGs will require more digital flexibility to allow AI to deliver approved updates across web platforms, mobile tools, and EHR-embedded CDS systems.

## Clinical decision support and predictive modeling

One major advantage of LCPGs is their potential for seamless integration into CDS tools. Currently, busy physicians face a workflow barrier when they must spend time manually seeking out CPGs, so in high-volume, fast-paced clinical environments guidelines may go unreviewed. Additionally, in certain areas of cardiology, such as heart failure, well-documented gaps remain in implementation of guideline-directed medical therapies, possibly due to clinician inertia or ignorance.^7^ Addressing this by integrating LCPGs into EHR-based CDS has the potential to greatly improve adherence to updated guideline recommendations. For example, automated prompts aligned with guideline recommendations can facilitate treatment initiation and nudge physicians to start appropriate heart failure therapy promptly.^7^ As new updates to guidelines are made, the CDS tools update in parallel, enabling physicians to see them immediately, so lack of clinician awareness does not delay incorporation of the latest guidance into clinical practice. Finally, we recognize that many CPGs in cardiology are long, complex documents that are not necessarily conducive to efficient information retrieval at the bedside. In these cases, LCPG-based EHR CDS tools can significantly improve efficiency and facilitate better uptake of updated guideline recommendations.

In addition to their potential as CDS tools, LCPGs can also be coupled with predictive modeling. Risk-based decision-making is a key component of cardiovascular medicine, with numerous traditional risk scores used in routine clinical practice, including CHA_2_DS_2_-VASc, atherosclerotic cardiovascular disease, and Global Registry of Acute Coronary Events 2.0. New risk stratification measures appear frequently and make their way into practice guidelines, as illustrated by the recently developed PREVENT (Predicting Risk of cardiovascular disease EVENTs) score, now recommended in the 2025 High Blood Pressure Guideline for the prevention, detection, evaluation, and management of patients in the United States.^8^ Keeping current on risk-assessment tools can be difficult both for CPG writers and for busy physicians, but LCPGs would address this by updating dynamically as new risk assessment tools are validated, allowing rapid integration of new scores, thresholds, and clinical variables into patient care. With the integration of LCPGs into EHRs, care can be personalized to each patient and their unique risk factors, ultimately enabling more precise interventions. Suboptimal utilization of risk assessment tools for quantitative cardiovascular disease risk for hypertension management in contemporary primary care is well-documented,^9^ but easier application of risk stratification is possible when LCPGs are integrated into EHRs.

## Democratization of knowledge

Limited access to guideline recommendations at the point of care has been cited as a major barrier to guideline adherence.^10^ Whereas access to traditional CPGs requires physicians to download PDFs, search through society portals, or even have a journal subscription, LCPGs can be disseminated in open access and web-based formats, or even mobile applications, promoting improved ease of use and accessibility in various geographic and resource settings. Care providers working in areas with limited resources may not have the training or background to interpret complex CPGs.^10^ Therefore, as guidelines increase in length and utilize increasingly complex methodology including systematic reviews, meta-analyses displaying results as forest plots and other unusual visuals, providers may be less likely to rely on them. LCPGs, especially when linked to EHRs and when paired with summary tables and predictive tools, reduce the cognitive and interpretative burden placed on physicians, further improving access of guideline recommendations to all providers, irrespective of background and training.

## Challenges

Despite the theoretical benefits of LCPGs, several challenges must be overcome before they can be widely accepted. First, significant infrastructure is required to develop the continuous monitoring tools required for the living systematic reviews that are foundational for quality LCPGs. Second, as with other AI medical applications, strict human oversight of the evidence generation and guideline updating processes would be required. Panels responsible for this would need to be nimble, and adapt their workflow to suit more continuous than episodic surveillance. Third, a major shift in the dissemination of CPGs from a PDF format would be required, and digitalized platforms for dissemination and updating must be developed. Fourth, it is paramount that implementation strategies are carefully and intentionally designed to avoid further widening disparities in cardiovascular outcomes with LCPGs between high-resource areas and those that may lack continuous internet access and the digital infrastructure required for LCPGs.

## Conclusions

Rigorous planning and efforts to develop the proper digital infrastructure are required before LCPGs can become a reality. Nevertheless, the opportunities offered by LCPGs to improve guideline adherence, personalize patient care with guideline-based point-of-care tools, and widely disseminate guideline recommendations are well worth the effort.

## Funding support and author disclosures

The authors have reported that they have no relationships relevant to the contents of this paper to disclose.
